# The Zucker Diabetic Fatty Rat as a Model for Vascular Changes in Diabetic Kidney Disease: Characterising Hydronephrosis

**DOI:** 10.3390/diagnostics15060782

**Published:** 2025-03-20

**Authors:** Amy McDermott, Nathalie Sarup Panduro, Iman Taghavi, Hans Martin Kjer, Stinne Byrholdt Søgaard, Michael Bachmann Nielsen, Jørgen Arendt Jensen, Charlotte Mehlin Sørensen

**Affiliations:** 1 Department of Biomedical Sciences, University of Copenhagen, 2200 Copenhagen, Denmark; stinnebyrholdt@gmail.com (S.B.S.); cmehlin@sund.ku.dk (C.M.S.); 2 Department of Diagnostic Radiology, Rigshospitalet, 2100 Copenhagen, Denmark; nathalie.sarup.panduro.01@regionh.dk; 3 Center for Fast Ultrasound Imaging, Department of Health Technology, Technical University of Denmark, 2800 Kongens Lyngby, Denmark; taghavi.it@gmail.com (I.T.); jaje@dtu.dk (J.A.J.); 4 Department of Applied Mathematics and Computer Science, Technical University of Denmark, 2800 Kongens Lyngby, Denmark; hmkj@dtu.dk; 5 Department of Clinical Medicine, University of Copenhagen, 2200 Copenhagen, Denmark

**Keywords:** diabetic kidney disease, type 2 diabetes, renal imaging, μCT, hydronephrosis, Zucker Diabetic Fatty rat

## Abstract

**Background/Objectives:** Diabetic kidney disease (DKD) is a significant concern for global healthcare, particularly in individuals with diabetes. The Zucker rat strain is a commonly used model of type 2 diabetes, despite awareness that this animal can develop hydronephrosis. In this study, we present novel imaging data evaluating the accuracy of this animal model in replicating the vascular aspects of human DKD while examining the impact of hydronephrosis on its validity as a disease model. **Methods:** This study reused data from a population of male Zucker Diabetic Fatty (ZDF; *n* = 22) rats and Zucker Lean (ZL) rats (*n* = 22) aged 12 to approximately 40 weeks. Vascular casting was performed to enable visualisation of the renal vasculature. Anatomical regional volumes and vascular density data were obtained from μCT scans using image thresholding and manual analysis. The effects of hydronephrosis were evaluated using renal functional parameters and histological examination. **Results:** A significantly lower cortical vascular density, as well as lower total renal vascular density, was seen in ZDF rats compared to ZL rats, independent of age. We identified that hydronephrosis affected 92% of ZDF rats and 69% of ZL rats. Hydronephrosis cavity size was significantly correlated with the degree of hyperglycaemia and rate of diuresis but had no other detected impact on renal function, vascularity, or tissue histological architecture. **Conclusions:** These findings support using the Zucker rat strain as a model for vascular changes in DKD. Despite identifying severe hydronephrosis in this population, it had minimal quantifiable impact on renal function or diabetes modelling.

## 1. Introduction

Diabetic kidney disease (DKD) is a major complication of diabetes and poses a significant global healthcare challenge, affecting 20–40% of individuals with diabetes [1]. DKD develops asymptomatically and is characterised by progressive damage to the renal vascular and tubular systems caused by autophagy, oxidative stress, and inflammatory processes [2]. Key risk factors include poor glucose regulation, hypertension, and genetic predisposition. DKD develops and progresses asymptomatically with a pathological process that is difficult to detect clinically until the onset of reduced estimated glomerular filtration rate (eGFR) and persistent albuminuria [3]. Despite its prevalence and impact, there is no consensus on which animal model best replicates the pathological features of DKD, particularly its vascular aspects [4].

The Zucker rat strain, including the inbred Zucker Diabetic Fatty (ZDF) rat, is widely used in type 2 diabetes research due to its monogenic loss-of-function mutation in the leptin receptor gene (fa, chromosome 5). This mutation leads to obesity, hyperphagia, and insulin resistance [5]. Zucker rats provide an established model for studying the progression from prediabetic to diabetic states, with closely related strains such as the Zucker Lean (ZL) rat providing a healthy phenotype for comparative studies [6,7]. However, the ZDF rat is also prone to hydronephrosis, which can alter renal anatomy and potentially affect its suitability as a model for DKD [8,9,10,11].

In this study, we build upon the dataset published by Søgaard et al., 2023 [12], which provided key insights into diabetic-related renal vascular damage in ZDF rats. Søgaard et al. hypothesised that the ZDF rat experiences renal microvascular damage early in DKD development, resulting in a lower vascular density than the non-diabetic Zucker Lean (ZL) rat. Their study utilised two-dimensional super-resolution renal imaging and preliminary μCT data, identifying data supporting their hypothesis. However, this imaging technique was limited in its ability to assess the entire renal vasculature, and their identification of hydronephrosis affecting both ZDF and ZL rats raised questions about the impact this condition may have on the vascular modelling of DKD.

Other research groups have identified hydronephrosis in several sub-strains of Zucker rats, including the ZDF, Zucker Lean, and Zucker Obese strains [8,9,10,11]. To date, no advanced imaging characterisation of hydronephrosis in these rats has been completed. Consequently, the full impact of hydronephrosis on the renal vasculature and DKD modelling remains unknown. There is also no consensus in the literature regarding the age of hydronephrosis onset, with some researchers finding animals as old as 12 weeks to be unaffected [13]. Hydronephrosis is defined as dilation of the renal pelvis and/or calyces. In humans, obstructive hydronephrosis is the most common presentation, often resulting from calculi, pregnancy, or neoplastic obstruction. Treatment for these conditions centres on the removal of the obstruction [14]. Non-obstructive hydronephrosis is usually grouped under the terms of congenital (dependent on the age at diagnosis) or iatrogenic hydronephrosis. These umbrella terms encompass several under-researched pathological aetiologies, including ureteropelvic junction obstruction, vesicoureteral reflux, ectopic ureter, megaureter, neurogenic bladder, posterior urethral valves, and ureterovesical junction obstruction [15,16,17]. While hydronephrosis has no reported developmental association with DKD or type 2 diabetes in humans, when it occurs concurrently, the condition can significantly impact the health of renal tissue and vasculature [18,19]. Although hydronephrosis is known to affect Zucker rats, its underlying aetiology remains unclear, and no research to date has determined its impact on diabetes modelling. This study addresses this gap by testing the hypothesis that hydronephrosis induces significant renal changes, thereby affecting its suitability as a model for DKD.

In this study, we present a comprehensive characterisation of the ZDF rat model for diabetic kidney disease (DKD) using advanced three-dimensional imaging techniques. Our analysis includes a detailed evaluation of renal anatomy, the hydronephrotic cavity, and vasculature, offering novel insights into how closely this model replicates the vascular features of human DKD. Additionally, we investigate the effects of hydronephrosis on kidney tissue, vasculature, and systemic health over time while exploring its potential link to diabetes progression. These findings provide valuable guidance for the selection and application of animal models in diabetes and DKD research, advancing both scientific understanding and translational relevance.

## 2. Materials and Methods

### 2.1. Data Collection

Full details of the experimental methods are published in Søgaard et al., 2023 [12]. Details relevant to this study are summarised here. Initial experiments used a total population of 44 rats (Charles River Laboratories^©^, Saint Germain, France) comprising two sub-strains: 22 male ZDF rats (strain 370, obese fa/fa) and 22 male ZL rats (strain 380, Lean fa/+). All rats were obtained between five and nine weeks of age and were housed in the Copenhagen University animal facility within the Department of Experimental Medicine. Animals were fed Purina 5008 (LabDiet, St. Louis, MO, USA) ad lib, water ad lib, and housed in the NexGen™ Rat Combo, 1800 cm^2^ reusable IVG housing system. All experiments presented in this paper were terminal. Experiments were conducted with three age groupings of 12 weeks, 22 weeks, and ≈40 weeks old. These time points correspond with known stages of disease progression: the development of hyperglycaemia by 12 weeks, early stage DKD prior to a significant increase in albumin-creatinine ratio (ACR) at 22 weeks, and late stage DKD with marked glomerulopathy at 40 weeks. Experimental groupings were not pre-allocated or randomised. Researchers were not blinded. After being transported from the animal facility to the experimental laboratories, all animals underwent a health assessment. Animals determined by veterinary evaluation to have compromised welfare were humanely euthanized.

Before anaesthesia, blood was collected from the tail vein, and glucose was measured using the Accu-Chek Aviva Blood Glucose Monitoring System (Roche Diabetes Care, Inc., Indianapolis, IN, USA). Anaesthesia was induced within an induction chamber (5% isoflurane, 65% nitrogen, 35% oxygen) and maintained on inhaled anaesthesia via a face mask (2% isoflurane, 65% nitrogen, 35% oxygen). The left jugular vein and left carotid artery were catheterised, and an endotracheal tube was placed by tracheostomy. The animal was ventilated (Ugo Basile, Gemonio, Italy) at 69 breaths per minute (2% isoflurane, 65% nitrogen, 35% oxygen). To prevent independent respiration, a 400 µL bolus of 85 mg/mL Nimbex (GlaxoSmithKline, London, UK) was given, followed by a constant rate infusion at 20 μL/min. Mean arterial pressure (MAP) during anaesthesia was monitored via the carotid artery using a Statham P23-dB pressure transducer (Gould, Oxnard, CA, USA) and LabChart (AD Instruments, Sydney, Australia). Through a midline laparotomy, a catheter was placed within the left ureter, and urine was collected over 30 min. Arterial blood and urine samples were collected to allow measurement of the following kidney function markers: creatinine clearance, albumin-to-creatinine ratio, and plasma urea. These samples were analysed using a commercial enzymatic kit in duplicate (CREP2, Ref. 03263991, Roche Diagnostics, Basal, Switzerland) and an Enzyme-Linked Immunosorbent Assay (ELISA) (Kit ab108789, Abcam, Cambridge, UK).

The left kidney was surgically exposed and imaged with super-resolution ultrasound imaging over 10 min to attain microvascular images, published by Søgaard et al., 2023 [12]. Then, the right kidney was excised, weighed, decapsulated, and fixed in 4% formaldehyde over 24 h for histology analysis. Following the right kidney excision, 1000 IE/kg of Heparin (1000 IE/mL, Amgros, Copenhagen, Denmark) was administered via the jugular catheter. The left kidney was then flushed with 9 mL of preheated (40°) heparinised saline 10 IE/mL at a rate of 2 mL/min. The animal was then euthanised by decapitation, and the kidney was infused with 3 mL of the contrast agent Microfil (Flow Tech Inc., Colorado, AZ, USA) at a rate of 1 mL/min. The contrast cured over 60 min, and then the kidney was excised, decapsulated, and fixed in 4% formaldehyde over 24 h before being embedded in paraffin. The kidney was scanned for 11 h in a ZEISS XRadia 410 Vera μCT scanner (Carl Zeiss Microscopy GmbH, Jena, Germany) using tube voltages of 40 kV, a power of 10 W, a 0.175 mA current, an appertaining LE2 filter, and a 360° scan around the vertical axis with 3201 different projections (0.112° rotation steps), at 0.49° angular increments. Full μCT volumes were reconstructed at an isotropic voxel size of 26.5 μm with voxel radiopacity represented by a 16-bit grey-scale value. Scans were then visually assessed, and where full contrast agent filling of the vascular system was not achieved, the individual was excluded from this dataset.

### 2.2. Image Segmentation and Vascular Density Measurement

**Figure 1:**
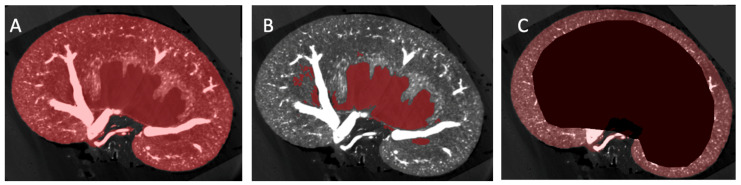
The methodology for generating region of interest (ROI) maps for anatomical analysis. ROI maps (**A**–**C**) were created to delineate three anatomical regions for volume and vascular density measurements. (**A**) ROI A: whole kidney. (**B**) ROI B: the pelvic cavity. (**C**) ROI C: renal cortex as delineated by the boundaries of the arcuate arteries and the kidney capsule. The three ROIs are overlaid here in red on the same μCT imaging plane. Three-dimensional regions of interest (ROIs) were manually generated from μCT kidney volumes using ITK-SNAP. ROI outlines were carefully delineated on every 4th to 6th μCT frame and subsequently merged into continuous 3D structures using the ‘Active Contour’ function.

The μCT volumes were processed using ITK-SNAP (version 4.0.1), and the “polygon” function was used to manually isolate 3D anatomical regions of interest [20]. Three regions of interest (ROI) were chosen: ROI A: the entire kidney, ROI B: the pelvic cavity (that may encompass a hydronephrotic cavity, if present), and ROI C: the cortex (defined by the boundary of the arcuate arteries and the kidney capsule) (Figure 1). Masks were created collaboratively by two trained individuals, a medical doctor and a veterinarian. Inter-rater variability was statistically analysed for two test scans, and no significant difference between selected areas included in anatomical masks was seen (Supplementary Data Figure S1). The absolute volume of the kidney and pelvic cavity was collected from ITK-SNAP and used to calculate the percentage of kidney tissue affected by hydronephrosis. Intensity threshold masks to isolate vascular tissue highlighted by the intravascular contrast agent Microfil were created and tailored for each kidney, considering the varying voxel radiopacity range (Supplementary Data Table S1). Vascular threshold masks were visually inspected by a doctor, veterinarian, and kidney physiology specialist for accuracy of vascular selection. Vascular density was calculated as the volume of the vascular threshold mask to the remaining volume of the kidney within anatomical regions of interest. Vascular density was measured in three anatomical regions: the full kidney (Figure 1, ROI A), the renal vascular tissue (ROI A minus ROI B), and the cortex (ROI C). A healthy renal pelvis size (non-hydronephrotic) was considered to be less than 5% of total kidney volume [21].

### 2.3. Renal Histology

**Figure 2:**
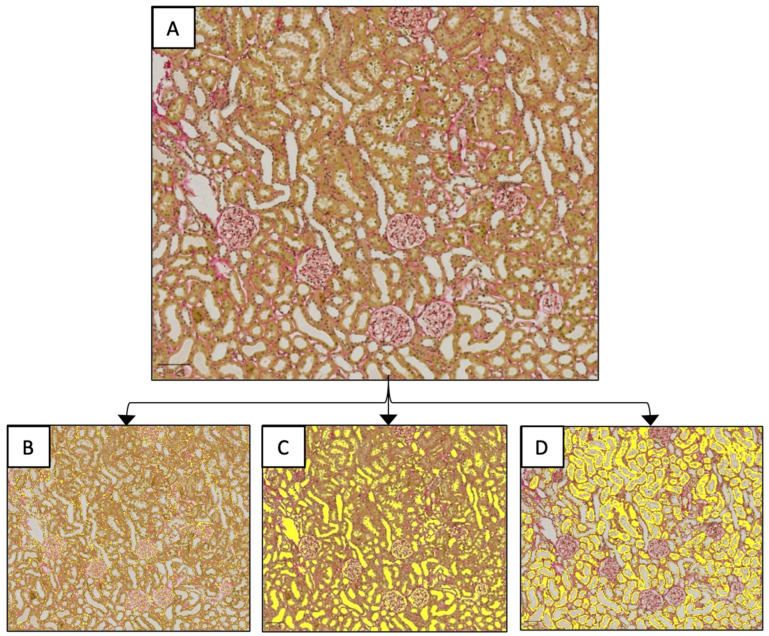
Method for stain deconvolution and colour-based segmentation. This approach was used to separate three categories of interest (**B**–**D**) from a single histological slide (**A**) using colour selection. (**A**) The ROI before colour selection. (**B**) ROI with selected areas of fibrosis overlaid in yellow. (**C**) ROI with selected areas of lumen overlaid in yellow. (**D**) ROI with selected areas of tubular tissue overlaid in yellow. All images were generated in Image-Pro 9.2 software.

Histological analysis was used to compare the impact of hydronephrosis on the renal tissue architecture of the ZL rat. There were not enough ZDF rats unaffected by hydronephrosis to provide a statistical control group, consequently, ZDF rats were not analysed histologically. The tissue plane for mounting was selected by an impartial histologist based on the best tissue preservations. Slides were stained with Sirius Red to enable visualisation of fibrosis and digitised using the AxioScan Z1 at a scale of X10. All histological analysis was completed using the software Image-Pro 9.2 software (Media Cybernetics). An ROI of approximately 0.3 mm^2^ of the renal cortex was selected, and stain deconvolution was used to identify the degree of fibrosis and proportion of luminal and tubular space (Figure 2).

### 2.4. Statistical Analysis

Throughout this paper, the threshold for statistical significance is set at *p* < 0.05. All statistical analysis and data visualisation was completed using R-Studio (version 4.3.1). Statistical analyses were conducted using one-way or two-way analysis of variance (ANOVA), depending on the experimental design, to assess differences among the three age groups alone or across the two strains. Tukey’s HSD post hoc test was applied to identify pairwise differences between groups. Additionally, to assess the impact of hydronephrosis and age on the renal tissue structure, a multivariate analysis of variance (MANOVA) was performed. MANOVA was used to evaluate the combined effects of hydronephrosis and age on the three assessed tissue types in the cortex: tubules, fibrosis, and lumen. When data is presented as a boxplot, the box represents the interquartile range (IQR) of the data, the horizontal line shows the median value, and the whiskers extend to the maximal range of the data limited to 1.5 times the IQR. In all statistical reporting and data visualisation, a *p*-value less than 0.05 is represented with one asterisk (*), less than 0.01 with two asterisks (**), and less than 0.001 with three asterisks (***).

## 3. Results

### 3.1. Study Population

This study commenced with a population of *N* = 44, with *n* = 22 of both male ZDF and male ZL rats. The following exclusions were made due to animal health: compromised welfare during pre-scan veterinary health assessment (*n* = 5; 4 ZDF and 1 ZL), rapid weight loss (*n* = 1; ZDF), rapid blood pressure drop during scanning (*n* = 1; ZL), on welfare grounds following husbandry issues with a blocked water feeder (*n* = 1; ZDF). Individuals were also removed when incomplete contrast agent vascular filling was observed on μCT assessment (*n* = 7; 3 ZDF and 4 ZL). Consequently, the final group sizes were: 13 subjects in the ZDF group (age 12, *n* = 4; age 22, *n* = 4; age ≈ 40, *n* = 5), and 16 subjects in the ZL group (age 12, *n* = 4; age 22, *n* = 5; age ≈ 40, *n* = 7).

### 3.2. Vascular Modelling of Diabetic Kidney Disease

**Figure 3:**
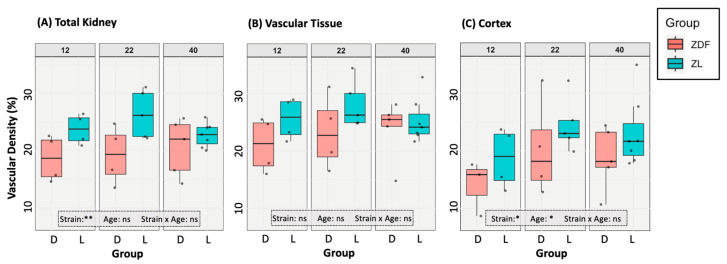
The effect of diabetes on regional vascular density in the investigated ROIs: (**A**) the total kidney, (**B**) the renal vascular tissue (pelvic cavity excluded), and (**C**) the Cortex. The rat strain, D = ZDF or L = ZL, is shown by the x-axis. Data points for individuals are displayed on the plot. Two-way ANOVA results are overlaid on each graph with non-significant interactions shown with “ns”. No significant pairwise comparisons exist in this data, so no significance bars are displayed.

The effects of rat age and strain on the vascular densities within the ROIs were analysed using two-way ANOVAs with Tukey’s HSD post hoc testing. In the analysis of the full kidney region, a significant main effect of rat strain was observed, with ZDF rats (D) showing significantly lower vascular densities than ZL rats (L) across all ages (F(1,23) = 9.99, *p* = 0.00463 **) (Figure 3A). However, no significant effects of age or the interaction between strain and age were found (age: *p* = 0.54; strain:age: *p* = 0.35). No significant pairwise comparisons were detected. When the vascular density of the remaining renal functional tissue was analysed, no significant effects of strain, age, or their interaction were detected (strain: *p* = 0.063; age: *p* = 0.52; strain:age: *p* = 0.68) (Figure 3B). No significant pairwise comparisons were detected. In the cortex region, a significant main effect of strain was found (F(1, 23) = 4.86, *p* = 0.038 *), indicating lower overall densities in ZDF rats compared to ZL rats (Figure 3C). A significant main effect of age was also detected (F(2, 23) = 3.46, *p* = 0.049 *). No significant interaction between strain and age was observed (*p* = 0.92). No significant pairwise comparisons were detected on post-hoc analysis or with *t*-testing of age-matched groups.

### 3.3. Hydronephrosis Prevalence and Severity

**Figure 4:**
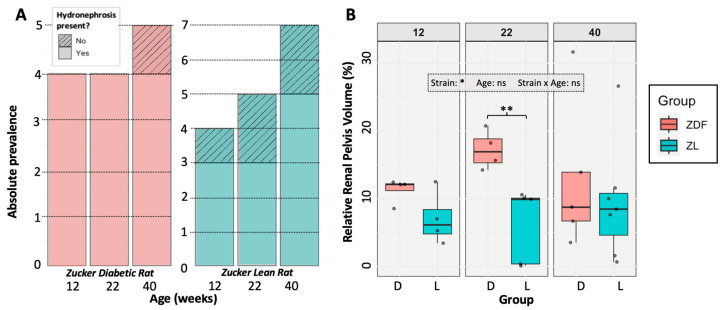
Hydronephrosis prevalence (**A**) and severity (**B**) in the ZDF and ZL populations. (**A**) Stacked boxplot displaying the prevalence of hydronephrosis (defined as a pelvic cavity exceeding 5% of the total renal volume) by rat sub-strain. (**B**) A box plot displaying the variation in size of the renal pelvic cavity across the population. Two-way ANOVA results are overlaid on the graphs with non-significant interactions shown with “ns”. Significant age-matched *t*-test results are displayed with significance bars.

At the point of euthanasia, a hydronephrotic pelvic cavity (exceeding 5% of the total kidney volume) was detected in 92% of ZDF rats and 69% of ZL rats, which is not a significantly different frequency (Fishers; *p* = 0.183) (Figure 4A). When comparing all rats, the pelvic cavity was, on average, significantly larger in the ZDF rats, at 13.7% of kidney volume, compared to a mean of 7.87% in ZL rats (*t*-test; *p* = 0.0308 *). A two-way ANOVA was used to examine the effects and interactions between rat age and strain (ZDF vs. ZL) on relative renal pelvic size in the affected rats. While rat strain had a significant impact on renal pelvic size (F(1,23) = 5.19, *p* = 0.0324 *), neither age nor any interaction between age and strain had a significant effect (age: *p* = 0.824; strain:age: *p* = 0.453). No significant pairwise differences were found on ANOVA post hoc analysis. The only significant difference on *t*-testing of matched age groups was at 22 weeks (*p* = 0.00753 **), with higher relative renal pelvis size in the ZDF rat (Figure 4B). Within the population affected by hydronephrosis, there was no significant difference in relative pelvic cavity size due to age, strain, or an interaction between them (strain: *p* = 0.157; age: *p* = 0.396; strain:age: *p* = 0.759). There were also no significant pairwise differences detected.

### 3.4. The Impact of Hydronephrosis on Renal Function and Anatomy

We analysed the impact of hydronephrosis on renal anatomy and function. As only one ZDF rat was not affected by hydronephrosis, this group was excluded from the statistical analysis. Consequently, the following analysis is limited to the ZL population.

#### 3.4.1. Renal Vascularity

**Figure 5:**
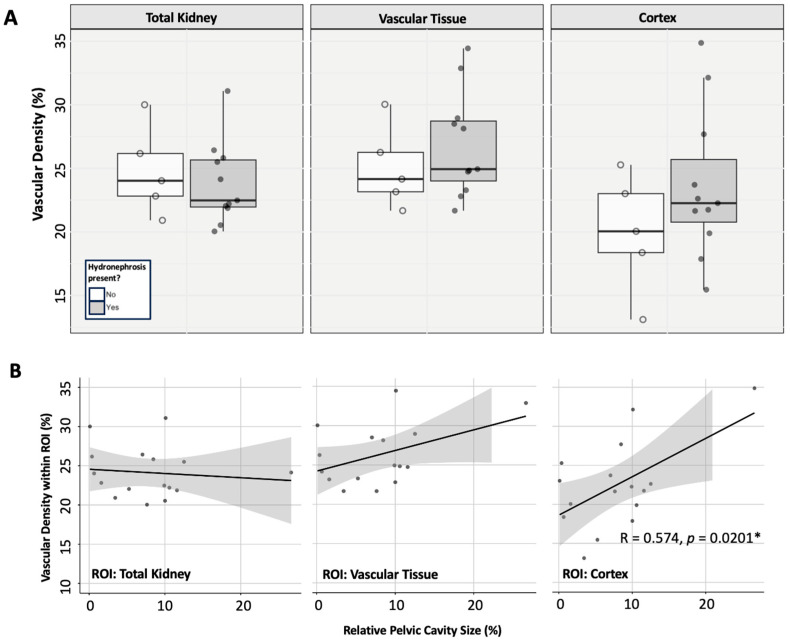
The impact of hydronephrosis on the renal vasculature in non-diabetic, ZL rats. (**A**) Differences in regional vascular density in rats affected or unaffected by hydronephrosis. (**B**) The linear relationship between relative renal pelvic cavity size and regional vascular density in ZL rats. Data points for individuals are displayed on the plots. Significant results are displayed.

To examine whether vascular density may be affected by the presence of hydronephrosis, we examined the ZL population. One-way ANOVA analysis showed that the age of the ZL rats had no significant impact on vascular density in any of the ROIs investigated: total renal vasculature (*p* = 0.147), remaining vascular tissue (*p* = 0.473), or within the cortex (*p* = 0.296). Thus, we proceeded to analyse the impact of hydronephrosis on the total ZL population, regardless of age. When exploring the effect of hydronephrosis (presence vs. absence) in the ZL group, there were no significant impacts on vascular density in any of the investigated ROIs: total renal vasculature (*t*-test; *p* = 0.619), remaining renal vascular tissue (*t*-test; *p* = 0.375), or the renal cortex (*t*-test; *p* = 0.210) (Figure 5A).

When examining the linear relationship between renal pelvic size and regional vascular density, no significant relationship was seen in the total kidney (Pearsons; R = −0.110, *p* = 0.685) or the remaining renal vascular tissue (Pearsons; R = 0.445, *p* = 0.0841) (Figure 5B). A significant linear relationship was seen between vascular density in the cortex and the relative size of the pelvic cavity (Pearsons; R = 0.574, *p* = 0.0202 *) (Figure 5B). It should be noted that if the outlier value is removed from the dataset, vascular density in the cortex and the relative size of the pelvic cavity would have a weak-positive non-significant correlation (Pearsons; R = 0.238, *p* = 0.393). However, we have no reason to defend the removal of this individual from the dataset, so we have completed all analyses with its inclusion.

#### 3.4.2. Histological Tissue Architecture

To assess the impact of hydronephrosis and age on the ZL rats’ renal tissue architecture, a MANOVA was conducted. This assessed these factors’ effects on three inter-related tissue types in the cortex: tubules, fibrosis, and lumen. Neither age (Pillai = 0.618, *p* = 0.290), hydronephrosis (Pillai = 0.238, *p* = 0.513), nor their interaction (Pillai = 0.419, *p* = 0.585) had a significant effect on the combined measured variables. Further, univariate ANOVA analysis showed hydronephrosis presence vs. absence not to affect any of the analysed tissue types when analysed in isolation (tubular tissue: *p* = 0.735; fibrosis: *p* = 0.261; luminal space: *p* = 0.184).

#### 3.4.3. Renal Functional Parameters

The following physiological variables measured in this study were all significantly affected by either age or rat strain: body weight, kidney weight, blood glucose, blood pressure, creatinine clearance, ACR, plasma urea, and rate of diuresis. These results are outside of the scope of this study; full details can be found in Supplementary Figure S2 and Supplementary Table S2.

To examine the impact of hydronephrosis, independent from the effects of diabetes, the ZL rat population was subset and analysed using two-way ANOVAs. In this population, age had a significant impact on the following parameters: body weight (*p* = 1.36 × 10^−9^ ***), kidney weight (*p* = 0.0245 *), kidney volume (*p* = 0.00504 **), blood glucose (*p* = 0.0372 *), blood pressure (*p* = 0.00166 **), and plasma urea (*p* = 8.77 × 10^−6^ ***). Due to the limited sample size, we could not further examine the binary impact of hydronephrosis on these variables. However, none of these parameters significantly correlated with the severity of hydronephrosis, except kidney volume, which showed a significant positive correlation with relative renal pelvis size (Pearsons; R = 0.518, *p* = 0.0397 *). Age had no significant impact on any of the following parameters in the ZL rat population: rate of diuresis (ANOVA; *p* = 0.172), creatinine clearance (ANOVA; *p* = 0.779), and ACR (ANOVA; *p* = 0.852). Thus, the following analyses will directly compare the total ZL population, disregarding age. These analyses showed that hydronephrosis does not affect creatinine clearance (*t*-test; *p* = 0.668), ACR (*t*-test; *p* = 0.151), or rate of diuresis (*t*-test; *p* = 0.745).

**Figure 6:**
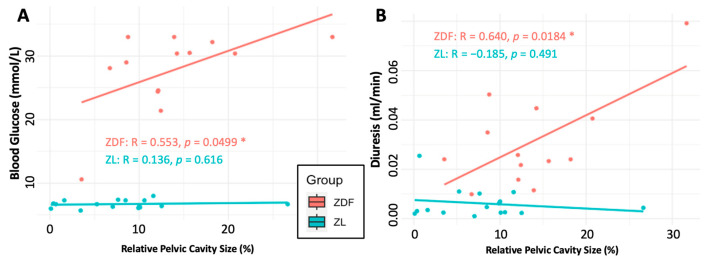
Scatter plot showing the linear relationship between pelvic cavity size and (**A**) blood glucose and (**B**) diuresis. Both plots are annotated with separate trend lines for the ZDF and ZL populations, as specified by the key. Correlation statistics for each sub-population are annotated.

When examining whether hydronephrosis affects diabetes modelling, we were unable to complete an ANOVA analysis due to the insufficient number of ZDF rats unaffected by hydronephrosis, which precluded statistical comparisons across groups. Consequently, the analysis was limited to methods that explore linear relationships and categorical associations. Only blood glucose levels and rate of diuresis showed any relationship to renal pelvis size. A strong positive, linear correlation was seen between higher blood glucose levels and a larger relative renal pelvis size (Pearsons; R = 0.509, *p* = 0.00477 **) (Figure 6A). The difference in correlation between the ZDF and ZL subsets was not statistically significant (Fisher’s r-to-z transformation; Z = −1.156, *p* = 0.248), despite a stronger positive correlation in the ZDF group (Pearsons; R = 0.553, *p* = 0.0499 *) than the ZL (Pearsons; R = 0.136, *p* = 0.616) (Figure 6A).

Across the entire population, a significant positive linear correlation was seen between higher relative renal pelvic cavity size and diuresis (Pearsons; R = 0.528, *p* = 0.00322 **). On further examination, the correlation between the larger relative size of the renal pelvis and increased rates of diuresis is isolated to the ZDF sub-population (Fishers; Z= −2.251, *p* = 0.0244 *). In the ZDF group, a positive correlation exists (Pearsons; R = 0.640, *p* = 0.0184 *), whereas there is no relationship between these variables in the ZL sub-population (Pearsons; R= −0.185, *p* = 0.491) (Figure 6B).

## 4. Discussion

**Figure 7:**
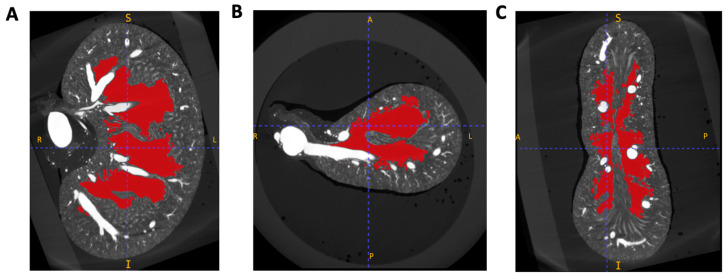
A μCT scan displaying a representative example of the anatomical structure of the hydronephrotic cavity in the Zucker Rat. Three matched views, (**A**) coronal, (**B**) axial, and (**C**) sagittal, of a ≈40-week-old ZDF rat kidney. The hydronephrotic cavity is overlaid in red. Images generated using ITK-SNAP (version 4.0.1).

This study investigated the renal anatomy of ZDF and ZL rats, characterising how diabetes affects renal vascular density whilst analysing the impacts of hydronephrosis, enabling assessment of the model’s appropriateness for DKD research. A lower vascular density in the total kidney and cortical region was observed in the total ZDF rat population compared to the ZL population, demonstrating similarities with human DKD (Figure 3). However, this reduction was not pronounced enough to result in significant pairwise differences at any of the study time points. We also identified a high prevalence of hydronephrosis within the population (92% in ZDF rats and 69% in ZL rats), resulting in an abnormally enlarged renal pelvis, with an average size of 14% in ZDF rats and 8% in ZL rats (Figure 4). Across the study population, the hydronephrotic cavity causes extensive reduction of the renal medullary tissue, following the existing bilobed shape of the rat renal pelvis (Figure 7). Despite this impact on the renal medullary tissue, hydronephrosis did not cause a significant reduction in total vascular density (Figure 5A). This degree of hydronephrosis has clear implications for the use of these animals in renal imaging studies, with significant distortion of the gross renal anatomy.

### 4.1. The Effects of Hydronephrosis on Healthy (ZL) Rats

In ZL rats, the development of hydronephrosis was not associated with any changes in the renal functional parameters measured in this study. Consequently, we can conclude that the hydronephrosis did not affect renal functionality to an extent that was clinically detectable at the investigated time points. We identified a positive correlation between relative pelvic cavity size and vascular density in the cortex, with the same trend visible but not statistically significant in the remaining renal vascular tissue (Figure 5B). There was no correlation between relative pelvic cavity size and total renal vascular density in the ZL rats. These data suggest that hydronephrosis has no detectable impact on the total renal vasculature, with the kidney able to compensate for hydronephrotic cavity expansion by preserving vasculature in the unaffected renal tissue, primarily in the cortex. We also identified that the increase in hydronephrotic cavity size occurs alongside an increase in total kidney volume but not weight. Consequently, we can conclude that hydronephrotic development is not an erosive process but that the kidney compensates for the increasing pelvic cavity size by expanding the total organ volume and preserving the existing vasculature into a smaller anatomical region of remaining vascular tissue. We hypothesise that the fluid within the abnormal renal pelvis exerts pressure on the surrounding tissues, resulting in the compression of the surrounding vascular tissue and some expansion of the organ capsule.

Histological assessment of the renal cortex in ZL rats showed no detectable changes in renal fibrosis, amount of luminal space, or tubular tissue related to the development of hydronephrosis. Hydronephrosis, which develops secondary to obstruction, chronic infectious, or inflammatory aetiologies, is commonly seen alongside severe renal fibrosis [22]. As we detected no change in renal fibrosis associated with hydronephrosis development, this reduces the likelihood of these aetiologies. We also detected no dilation of the renal tubular system, as is commonly reported in obstructive hydronephrosis. These findings, together with the normal rates of urination, enable us to remove obstructive causes from the likely aetiologies of hydronephrosis in this study population. Age did not have a significant impact on the size of the hydronephrotic cavity in either rat strain across the examined time points. This indicates that the hydronephrosis in these animals is non-progressive and does not become obstructive. A similar disease presentation is seen in children affected by congenital hydronephrosis [23]. Research has also shown that congenital hydronephrosis in mice does not cause the tubular morphological damage that is seen with surgically induced obstructive hydronephrosis [24]. This demonstrates that the kidney anatomy is less severely affected by chronic hydronephrosis. Therefore, the findings of this study, together with the knowledge of a high prevalence of hydronephrosis in several closely related rat sub-strains, suggest that this condition is congenital with a strong hereditary nature. However, further research, particularly utilising young animals, is needed to confirm this hypothesis.

### 4.2. Implications for Diabetes and DKD Research

While hydronephrosis undoubtedly affects this model’s usability, we successfully identified a significant reduction in cortex vascular density regardless of age in the ZDF rat compared to the ZL rat (Figure 3). This is an encouraging finding when considering the applicability of the ZDF sub-strain for DKD research, as microvascular damage is hypothesised to be one of the first pathogenic processes occurring in DKD in human patients. Our study confirmed that hydronephrosis in ZL rats does not lead to pathological changes in the cortical renal tissue architecture. Although we were unable to directly confirm this finding in ZDF rats, hydronephrosis likely follows a similar developmental trajectory in both populations. Therefore, our data suggest that hydronephrosis in these models does not necessarily compromise their suitability for DKD research involving renal histological analyses.

Due to limitations in sample size, our ability to analyze the impact of hydronephrosis on the physiological expression of diabetes in ZDF rats was restricted, limiting our analysis to only linear relationships with renal pelvis size. We acknowledge that the assumption of linearity may oversimplify the dynamics of the relationships observed. Notably, this study revealed a significant positive correlation between larger relative pelvic (hydronephrotic) cavities and both the severity of hyperglycemia and rates of diuresis in ZDF rats—two closely interrelated variables relevant to diabetes research. It has been hypothesised that the increased severity of hydronephrosis in the ZDF rat may be due to an inability to shed glucose at the needed rate, contributing to a cycle of persistent and severe hyperglycemia and subsequent renal damage leading to increased hydronephrosis [8]. While this paper detected no statistical evidence of progressive hydronephrosis over the experimental time course, our findings provide further data on the relationship between hyperglycemia, diuresis, and hydronephrosis in the ZDF rat. This raises important questions for researchers using the degree of hyperglycemia as a marker of health or treatment response, as the hydronephrotic state may introduce some degree of variation into experimental results.

Importantly, while we found no evidence that hydronephrosis was associated with changes in renal functional parameters measured in this study, this does not preclude the possibility of alterations in urinary patterns, such as increased frequency, urgency, or reduced urination, which may be worth investigating further. The observed correlation between hydronephrosis and hyperglycemia appears to be indirect, suggesting no shared underlying mechanisms between the two conditions.

Additionally, we noted no evidence that hydronephrosis is a progressive condition in the ZDF strain, as it is likely to present congenitally or at a young age, before the onset of diabetes. This raises the possibility that hydronephrosis may be linked to metabolic disturbances unrelated to diabetes or hyperglycemia. Consequently, while the severity of hydronephrosis may influence the later expression of diabetes symptoms, this conclusion should be interpreted with caution, as further research is needed to elucidate the precise nature of this relationship and rule out confounding factors.

### 4.3. Limitations

With several animals excluded during the experiment and additional exclusions due to incomplete data, the reduced sample size and study power limit the strength and generalizability of the conclusions drawn from this research. A post-hoc power analysis, considering the final population sizes, comparing the vascular density results of the total ZL (*n* = 16) and ZDF (*n* = 13) populations gave an 87.2% post-hoc power. However, for all analyses where the populations were sub-set into age or dependent on hydronephrosis, the post-hoc power is below 80%. Consequently, drawing conclusions from this data should be done cautiously, and further study is indicated. With retrospective consideration, it is also possible that experimental bias was introduced in this study as rats were not systemically randomised into age groups at the start of the experiment. This, combined with the poor health of the rats within the study, may have led to the selection of the sickest rats at each time point, resulting in rats at ≈40 weeks being those with the best overall health. This theory may be supported by the fact that ZDF rats at ≈40 weeks had the lowest prevalence of hydronephrosis of the ZDF groups, significantly lower blood pressure than other time points, and lower body weight.

## 5. Conclusions

This study identified a reduction in total and cortical vascular density in diabetic ZDF rats compared to ZL rats, indicating that diabetes causes damage to the renal microvasculature, consistent with DKD. Additionally, our findings demonstrate that while hydronephrosis significantly affects renal gross anatomy, it has minimal impact on the vasculature, renal functional parameters, or histological tissue architecture. However, critical questions remain unanswered: why does hydronephrosis occur, why does it correlate with hyperglycemia, is it congenital, and can it be prevented? In humans, congenital non-obstructive hydronephrosis is a complex condition with poorly understood embryological origins and treatment strategies [23]. Addressing these questions requires further research. Nonetheless, this study offers a novel and comprehensive characterisation of renal health in these rats, providing valuable insights for selecting preclinical animal models in diabetes, renal imaging, and renal vascular research.

## Data Availability

Data available in a publicly accessible repository. The original data presented in the study are openly available in FigShare at DOI:10.6084/m9.figshare.28303718.

## Supplementary Materials

The following supporting information can be downloaded at: https://www.mdpi.com/article/10.3390/diagnostics15060782/s1, Figure S1: Describing inter-rater variability between manually created μCT volume masks for the full kidney and the renal pelvis between two blinded assessors; Figure S2: The effect of age and rat sub-strain on the measured physiological variables; Table S1: Intensity threshold values for each μCT volume, enabling the optimal selection of the renal vasculature; Table S2: Data analysis of the effect of age and rat sub-strain on the measured physiological variables in this study.

## Abbreviations

The following abbreviations are used in this manuscript:

DKD	Diabetic kidney disease
ZDF	Zucker Diabetic Fatty
ZL	Zucker Lean
ROI	Region of Interest
ANOVA	Analysis of Variance
MANOVA	Multivariate Analysis of Variance
IQR	Interquartile Range
