# Supplementary material for: The Zucker Diabetic Fatty Rat as a Model for Vascular Changes in Diabetic Kidney Disease: Characterising Hydronephrosis
Source: Diagnostics (Basel). 2025 Mar 20;15(6):782. doi: 10.3390/diagnostics15060782 (PMC11941088; doi:10.3390/diagnostics15060782)
Supplement: Supplementary file 1 [file diagnostics-15-00782-s001.zip › diagnostics-3476982-supplementary.pdf]

Evaluating the Zucker Diabetic Fatty Rat as a Model for Diabetic Kidney Disease: A Focus on Hydronephrosis

** Supplementary Data**

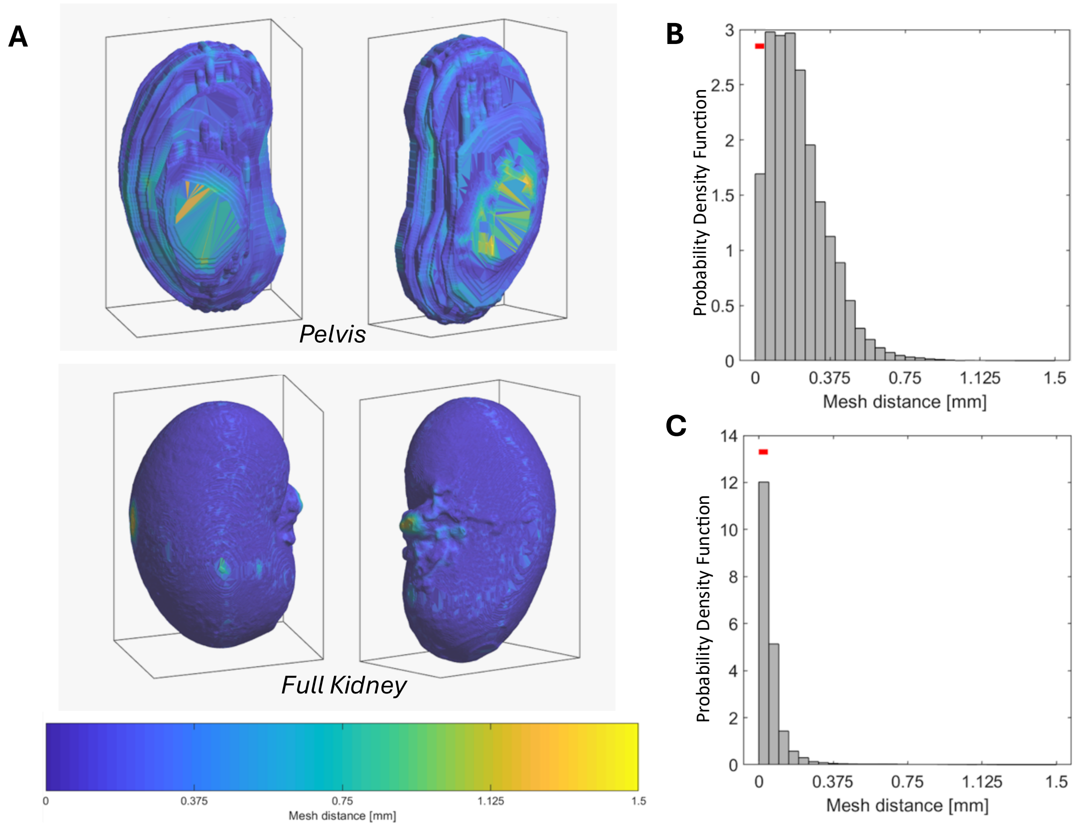

Supplementary Data: Figure S1: Describing inter-rater variability between manually created µCT volume masks for the full kidney and the renal pelvis between two blinded assessors. A) Visual representation of the degree of µCT mask disagreements. B, C) Histograms illustrating the distribution of disagreement distances between the 3D anatomical region masks of the B) renal pelvis and C) full kidney.

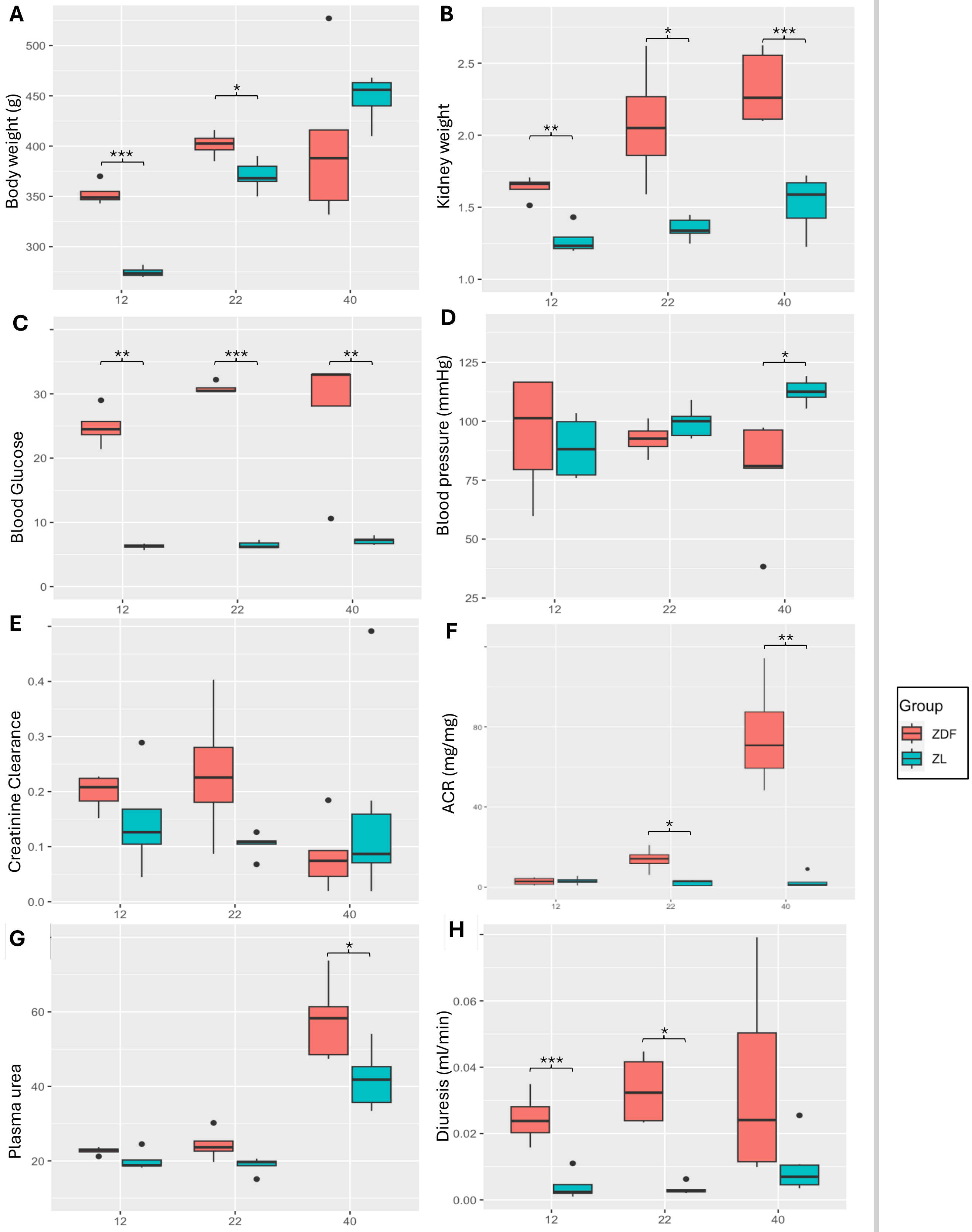

Supplementary Data Figure S2: The effect of age and rat sub-strain on the measured physiological variables. Values analysed are body weight (g), kidney weight (g), blood glucose, blood pressure, creatinine clearance (ml/min), ACR, plasma urea, and rate of diuresis. Portions of this data have been published previously in Figure 4 of Søogaard et al. (2023)[4].

Supplementary Data Table S1: Intensity threshold values for each µCT volume, enabling the optimal selection of the renal vasculature. Two blinded assessors completed the analysis separately and the average of their results was used as the final threshold value. The µCT voxel range for each reconstruction is included in the table.

Zucker Lean Rats					Zucker Diabetic Fatty Rats				
Rat ID	Voxel Range	Assessor 1	Assessor 2	Threshold	Rat ID	Voxel Range	Assessor 1	Assessor 2	Threshold
21	33,1 - 255	67,90	64,6	**66,3**	27	44,7 - 255	85,00	71,2	**78,1**
22	43,1 - 255	72,00	72,9	**72,5**	28	44,7 - 255	70,00	67,9	**69,0**
24	38,1 - 255	68,00	71,2	**69,6**	29	38,1 - 255	75,00	58,0	**66,5**
25	38,1 - 255	77,00	69,0	**73,0**	30	16,6 - 255	65,00	54,6	**59,8**
33	39,5 - 255	68,00	66,2	**67,1**	40	20 - 255	79,50	71,2	**75,4**
36	36,5 - 255	64,60	61,3	**63,0**	41	41,5 - 255	69,50	66,2	**67,9**
37	48 - 255	74,50	74,5	**74,5**	42	53 - 255	89,50	82,8	**86,2**
38	39,7 - 255	80,00	74,5	**77,3**	43	34,8 - 255	74,50	76,2	**75,4**
39	50 - 255	83,50	79,5	**81,5**	56	39,5 - 255	70,50	64,6	**67,6**
47	42 - 255	74,00	67,9	**71,0**	57	26,5 - 255	63,00	58,0	**60,5**
48	39 - 255	76,00	72,6	**74,3**	58	48 - 255	89,00	76,2	**82,6**
50	35 - 255	71,20	67,9	**69,6**	59	35 - 255	69,50	64,6	**67,1**
51	40 - 255	68,00	69,5	**68,8**	61	43 - 255	71,50	71,2	**71,4**
52	35 - 255	63,00	62,9	**63,0**					
54	25 - 255	69,50	62,9	**66,2**					
55	40 - 255	73,00	71,2	**72,1**					

Supplementary Data Table S2: Data analysis of the effect of age and rat sub-strain on the measured physiological variables in this study. Variables analysed are body weight (g), kidney weight (g), blood glucose, blood pressure, creatinine clearance (ml/min), ACR, plasma urea, and rate of diuresis.

	ANOVA			Impact of Strain Between Age-matched Goups (Welch T-Test)			
	Rat Sub-strain	Age	Strain:Age Interaction	Total Population	12	20	≈40
**Body Weight (g)**	ns	***p* =1.2e-06*****	***p* =0.00244****				
**Kidney Weight (g)**	***p* =6.68e-08*****	***p* =0.000426*****	ns		*p* =0.00211**<br>t=5.317, df=5.705<br>ZDF =1.64. ZL = 1.27	*p = 0.0414*<br>t = 3.332, df = 3.159<br>ZDF = 2.08, ZL = 1.35*	*p* =0.000469***<br>t = 6.173, df = 6.955<br>ZDF = 2.33, ZL = 1.53
**Blood Glucose**	***p* =2.73e-12*****	ns	ns	*p* =4.442e-08***<br>t = 11.924, df = 12.183<br>ZDF=27.739, ZL=6.713			
**Blood Pressure**	***p* =0.0182***	ns	***p* =0.0263***	*p* =0.0393<br>t = -2.2158, df = 18.779<br>ZDF=87.863, ZL=102.708			
**Creatinine Clearance**	ns	ns	ns				
**Albumin-Creatinine ratio**	***p* =1.21e-06*****	***p* =1.17e-05*****	***p* =1.98e-06*****		*ns*	*p* =0.0287*<br>t = 3.750, df = 3.260<br>ZDF = 138.446, ZL = 21.728	*p* =0.00550**<br>t = 5.408, df = 4.042<br>ZDF = 871.473, ZL = 24.987
**Plasma Urea**	***p* =0.00367****	***p* =2.53e-10*****	ns		*ns*	*ns*	*p* =0.0238*<br>t = 2.902, df = 6.772<br>ZDF = 57.880, ZL = 41.614
**Rate of Diuresis**	***p* =7.57e-05*****	ns	ns	*p* =0.000435***<br>t = 4.571, df = 14.01<br>ZDF=0.0312, ZL=0.00620			
